# A *GC* polymorphism associated with serum 25-hydroxyvitamin D level is a risk factor for hip fracture in Japanese patients with rheumatoid arthritis: 10-year follow-up of the Institute of Rheumatology, Rheumatoid Arthritis cohort study

**DOI:** 10.1186/ar4516

**Published:** 2014-03-20

**Authors:** Shinji Yoshida, Katsunori Ikari, Takefumi Furuya, Yoshiaki Toyama, Atsuo Taniguchi, Hisashi Yamanaka, Shigeki Momohara

**Affiliations:** 1 Institute of Rheumatology, Tokyo Women’s Medical University, 10-22 Kawada, Shinjuku, Tokyo 162-0054, Japan; 2 Department of Orthopaedic Surgery, Keio University School of Medicine, 35 Shinano, Shinjuku, Tokyo 160-8582, Japan

## Abstract

**Introduction:**

Vitamin D deficiency has been reported to be common in patients with rheumatoid arthritis (RA) who have a higher prevalence of osteoporosis and hip fracture than healthy individuals. Genetic variants affecting serum 25-hydroxyvitamin D (25(OH)D) concentration, an indicator of vitamin D status, were recently identified by genome-wide association studies of Caucasian populations. The purpose of this study was to validate the association and to test whether the serum 25(OH)D-linked genetic variants were associated with the occurrence of hip fracture in Japanese RA patients.

**Methods:**

DNA samples of 1,957 Japanese RA patients were obtained from the Institute of Rheumatology, Rheumatoid Arthritis (IORRA) cohort DNA collection. First, five single nucleotide polymorphisms (SNPs) that were reported to be associated with serum 25(OH)D concentration by genome-wide association studies were genotyped. The SNPs that showed a significant association with serum 25(OH)D level in the cross-sectional study were used in the longitudinal analysis of hip fracture risk. The genetic risk for hip fracture was determined by a multivariate Cox proportional hazards model in 1,957 patients with a maximum follow-up of 10 years (median, 8 years).

**Results:**

Multivariate linear regression analyses showed that rs2282679 in *GC* (the gene encoding group-specific component (vitamin D binding protein)) locus was significantly associated with lower serum 25(OH)D concentration (*P* = 8.1 × 10^-5^). A Cox proportional hazards model indicated that rs2282679 in *GC* was significantly associated with the occurrence of hip fracture in a recessive model (hazard ratio (95% confidence interval) = 2.52 (1.05-6.05), *P* = 0.039).

**Conclusions:**

A two-staged analysis demonstrated that rs2282679 in *GC* was associated with serum 25(OH)D concentration and could be a risk factor for hip fracture in Japanese RA patients.

## Introduction

Vitamin D regulates calcium and phosphate homeostasis and reportedly has other roles in human physiology [1,2]. Vitamin D deficiency is associated with the occurrence of osteoporosis, autoimmune diseases, cardiovascular disease, type 1 and type 2 diabetes mellitus, and several types of cancer [3-7]. Vitamin D also plays an important role in the maintenance of the musculoskeletal system. It is positively associated with muscle strength and physical performance, and is negatively associated with fall and fracture risk [8-11].

Vitamin D deficiency has been reported to be common in patients with rheumatoid arthritis (RA), and more than 70% of Japanese patients with RA had vitamin D deficiency [12,13]. Significant associations of vitamin D deficiency were found with some independent clinical risk factors: female gender, younger age, high disability score in the Japanese version of the Health Assessment Questionnaire (J-HAQ), low serum total protein level, low serum total cholesterol level, high serum alkaline phosphate (ALP) level, and use of non-steroidal anti-inflammatory drugs (NSAIDs) [12].

Bone mineral density (BMD) is the major predictor of osteoporotic fracture, and previous studies have reported that patients with RA have a lower BMD and are at greater risk of hip fracture than healthy individuals [14,15]. We have previously shown that a high J-HAQ disability score, advanced age, history of total knee replacement (TKR), and low body mass index (BMI) were clinical risk factors for the occurrence of hip fracture in Japanese patients with RA [16].

Prior twin and family studies suggested that genetic factors also influence serum vitamin D concentration [17,18]. Genetic variants that affect serum 25-hydroxyvitamin D (25(OH)D) concentration, an indicator of vitamin D status, were recently identified in a meta-analysis of genome-wide association studies (GWAS) in Caucasian populations [19,20]. Though the presence of heterogeneity in genes related to RA has been suggested in many population-based studies [21], these associations remain unknown in the Japanese population.

The purpose of this study was to validate the possible association between genetic variants and serum 25(OH)D concentration and to test whether the serum 25(OH)D-linked variants were associated with the occurrence of hip fracture in Japanese patients with RA.

## Methods

### Study population

This study was a part of the Institute of Rheumatology, Rheumatoid Arthritis cohort study (IORRA), a single-institution-based, large-scale prospective observational cohort study with an enrollment of over 5,000 Japanese patients with RA, began in 2000 [12,16,22]. DNA samples of 1,957 Japanese RA patients were obtained from the IORRA DNA collection. All the patients satisfied the American College of Rheumatology 1987 revised criteria for RA. Tokyo Women’s Medical University Genome Ethics Committee approved the present study and each individual signed an informed consent form after receiving a verbal explanation of the study.

### SNP selection and genotyping

Five single nucleotide polymorphisms (SNPs) were selected from the results of recent genome-wide association studies (GWAS) that showed positive associations between serum 25(OH)D levels and the following: rs2282679 in the *GC* (group-specific component) locus, rs3829251, rs12785878 and rs1790349 in the *DHCR7/NADSYN1* (7-dehydrocholesterol reductase/nicotinamide-adenine dinucleotide synthetase 1) locus, and rs10741657 in the *CYP2R1* (cytochrome P450, family 2, subfamily R, polypeptide 1) locus [19,20]. Genotyping was performed by using the TaqMan fluorogenic 5′ nuclease assay according to the manufacturer’s instructions (Applied Biosystems, Tokyo, Japan). Duplicate samples and negative controls were included to ensure accuracy of SNP genotyping. All polymerase chain reactions were performed by using the GeneAmp PCR System 9700 (Applied Biosystems), and endpoint fluorescent readings for TaqMan assays were done on an ABI PRISM 7900 HT Sequence Detection System (Applied Biosystems) as described elsewhere [22].

### Measurement of serum 25(OH)D concentration

Serum 25(OH)D concentrations were measured in 899 of 1,957 patients from whom we obtained DNA samples. As vitamin D is synthesized in the skin under the influence of sunlight and the seasonal variance in serum levels of vitamin D is well known, serum samples were collected in the same season, spring of 2011 [23]. The DiaSorin 25(OH)D^125^I radioimmunoassay kit was used for quantitative determination of serum 25(OH)D concentration.

### Assessment of hip fracture

The occurrence of hip fracture after enrollment in IORRA was determined by the response to a patient questionnaire every six months from October 2000 to October 2010 with a maximum follow-up period of 10 years. The data were confirmed by review of medical records and radiographs as described elsewhere [16]. Only the occurrence of the first hip fracture reported by patients was included in this study. The occurrence of hip fracture caused by major trauma such as car accidents was excluded. A total of 39 hip fractures in 39 patients were identified and included in this study.

### Statistical analyses

#### Stage 1: cross-sectional analyses of SNPs associated with serum 25(OH)D concentration

A two-staged analysis was used. First, cross-sectional associations were examined between serum 25(OH)D concentrations and risk alleles of each SNP using multivariate linear regression analyses (adjusted for independent non-genetic risk factors) in 899 of the 1,957 patients. The putative risk alleles were defined as the alleles that are associated with lower serum 25(OH)D concentration based on prior reports [19,20]. The number of risk alleles for genotyped SNPs (0, 1 and 2) was used to test the additive effect of the alleles on lower serum 25(OH)D concentration. The following factors that have been shown to be significantly associated with lower serum 25(OH)D levels were selected as independent non-genetic risk factors: gender, age, J-HAQ disability score, serum total protein level, serum total cholesterol level, serum ALP level, and NSAID use [12].

#### Stage 2: longitudinal association analyses of SNPs associated with occurrence of hip fracture

The SNP that showed a significant association with serum 25(OH)D concentration was selected to test the longitudinal association with the hip fracture event. The length of time from the date of enrollment in IORRA to the date of the occurrence of hip fracture was calculated. A multivariate Cox proportional hazards model adjusted for independent non-genetic risk factors that were associated with hip fracture was performed in the cohort of 1,957 patients [16]. The following factors at the time of hip fracture reported were used as the independent non-genetic risk factors: J-HAQ disability score, age, history of TKR, and BMI. The proportional hazards assumption for the Cox model was assessed using log-minus-log plots for survival analysis. All statistical analyses were performed using the R software package [24].

## Results

### Clinical and demographic characteristics of the patients

Demographic, clinical and therapeutic data describing the 1,957 patients at the time of enrollment in IORRA are shown in Table 1. In this cohort, serum 25(OH)D concentrations were measured in 899 patients in the spring of 2011 (Table 1). The median serum 25(OH)D concentration was 15.30 ng/mL (IQR, 12.10 to 20.10). During a maximum follow-up period of 10 years (median, 8.0 years; IQR, 4.5 to 10.0 years) 39 hip fractures in 1,957 patients were identified.

**Table 1:** Demographic, clinical and therapeutic data at the time of an enrollment in IORRA and at the time of measurement of serum 25(OH)D concentration

**Factor**	**At the time of enrollment in IORRA (n = 1957)**	**At the time of measurement of serum 25(OH)D concentration (spring 2011, n = 899)**
Age, years	57.5 (49.5 to 64.6)	64.3 (56.7 to 70.6)
Sex, female	1668 (85.2)	766 (87.7)
Duration of disease, years	7.0 (2.0 to 14.0)	16.0 (11.0 to 23.0)
BMI, kg/m^2^	21.2 (19.4 to 23.3)	21.2 (19.2 to 23.2)
DAS28	4.2 (3.3 to 5.0)	3.0 (2.4 to 3.8)
J-HAQ	0.8 (0.2 to 1.4)	0.6 (0.1 to 0.8)
RF, positive	1531 (81.8)	698 (80.0)
History of smoking, ever	641 (34.5)	235 (28.4)
History of TKR, ever	85 (4.3)	178 (20.4)
DMARDs use, ever	1670 (85.3)	777 (89.0)
Methotrexate use, ever	792 (40.8)	638 (73.1)
Biologic use, ever	12 (0.6)	155 (17.8)
Corticosteroid use, ever	932 (47.6)	399 (45.7)
Bisphosphonate use, ever	63 (3.2)	280 (32.1)
Active vitamin D use, ever	61 (3.1)	112 (12.8)
Serum total protein level, g/dL	Data not available	7.3 (7.0 to 7.6)
Serum total cholesterol level, mg/dL	Data not available	209.0 (188.0 to 231.0)
Serum alkaline phosphate level, IU/L	Data not available	258.0 (213.0 to 321.2)
NSAIDs use, ever	Data not available	515 (59.0)

Data are presented as median (IQR) or n (%). IORRA, Institute of Rheumatology, Rheumatoid Arthritis cohort; BMI, body mass index; DAS28, disease activity score in 28 joints; J-HAQ, Japanese version of the Health Assessment Questionnaire; RF, rheumatoid factor; TKR, total knee replacement; DMARD, disease modifying antirheumatic drug; NSAID, non-steroidal anti-inflammatory drug.

### Genotyping

The overall genotyping success rate was 97.3% and the genotype concordance rate was 100% as assessed by duplicate samples. After the application of quality control criteria for genotyping (removed samples that consistently failed for >20% (1/5) of SNPs, removed SNPs with a call rate <95% after removing samples that consistently failed), 1,915 of the 1,957 samples and all polymorphisms were accepted for the analyses. The studied polymorphisms were found to be in Hardy-Weinberg equilibrium.

### Stage 1: cross-sectional analyses of SNPs associated with serum 25(OH)D concentration

**Figure 1:**
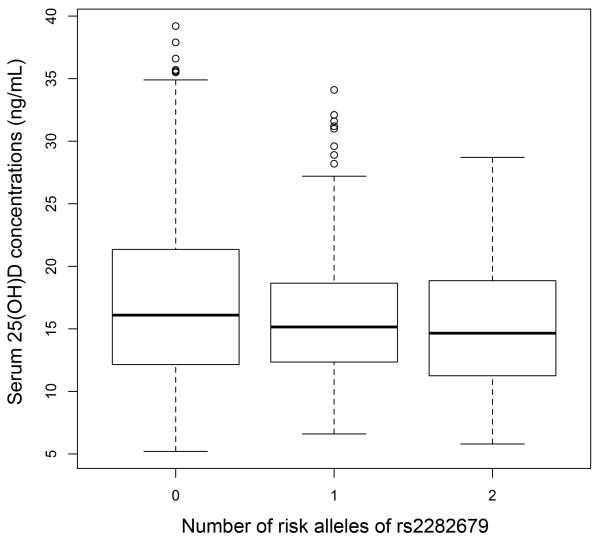
**Boxplots representing the distribution of serum 25(OH)D concentration according to the number of the risk allele of rs2282679 (minor allele, C) in the *GC *locus.** Each box represents the IQR range of values, with the bold line showing the median value. The vertical lines show maximum and minimum values that fall within 1.5 box lengths, the open circles show extreme values >1.5 box plot lengths.

A multivariate linear regression analysis adjusted for independent non-genetic risk factors showed that the minor allele of rs2282679 (= C) in *GC* was significantly associated with lower serum 25(OH)D concentrations (*P* = 8.1 × 10^-5^, Table 2). The median serum 25(OH)D concentrations (ng/mL) for the genotypes of rs2282679 in *GC* were 16.1, 15.2 and 14.7, respectively for AA, AC and CC (Figure 1). The other SNPs did not show a significant association. The minor allele frequency of rs2282679 (*GC*) in RA patients (= 0.259) did not differ significantly from a Japanese control population (= 0.258, *P* = 0.98 by chi-squared test, n = 752), which was obtained from the DNA collection of the Pharma SNP Consortium, Tokyo, Japan, currently entrusted to the Health Science Research Resources Bank, Osaka, Japan, as described elsewhere [22].

**Table 2:** Multivariate linear regression analyses of each SNP associated with serum 25(OH)D concentration

**Locus**	**SNP**	**MAF**^**†**^	**β**	**SE**	***P*-value**
*GC*	rs2282679	0.259 (A/C)	-0.13	0.033	8.1 × 10^-5^
*DHCR7/NADSYN1*	rs3829251	0.375 (G/A)	-0.0031	0.032	0.92
	rs12785878	0.328 (G/T)	0.0057	0.032	0.86
	rs1790349	0.361 (A/G)	-0.016	0.032	0.63
*CYP2R1*	rs10741657	0.387 (G/A)	0.035	0.034	0.30

All analyses were adjusted for independent non-genetic risk factors: gender, age, Japanese version of the Health Assessment Questionnaire disability score, total protein level, total cholesterol level, alkaline phosphate level, and non-steroidal anti-inflammatory drugs use [12]. ^†^Alleles are listed as major allele/minor allele. SNP, single nucleotide polymorphism; MAF, minor allele frequency; SE, standard error; *GC*, group-specific component; *DHCR7/NADSYN1*, 7-dehydrocholesterol reductase/nicotinamide-adenine dinucleotide synthetase 1; *CYP2R1*, cytochrome P450, family 2, subfamily R, polypeptide 1.

### Stage 2: longitudinal association analyses of SNPs associated with occurrence of hip fracture

**Figure 2:**
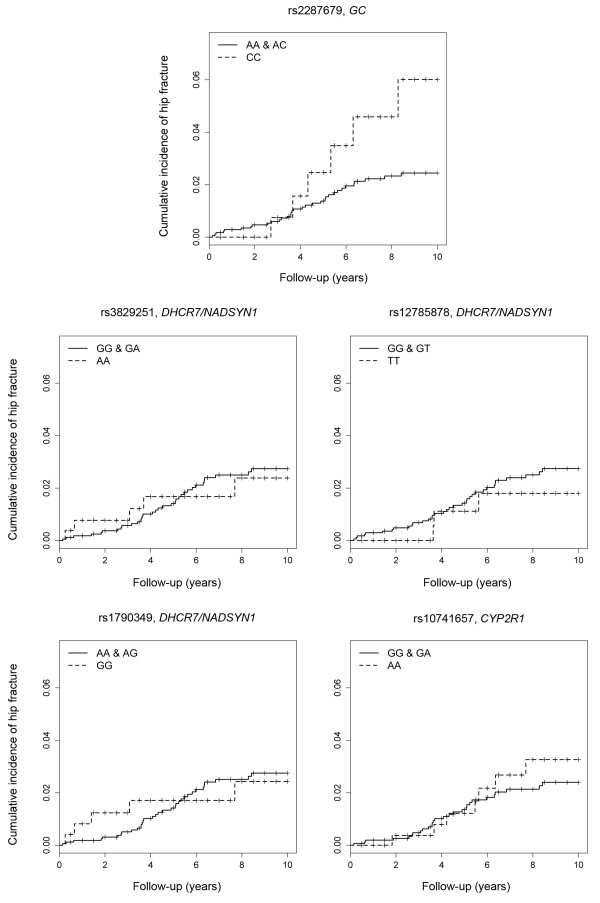
**Cumulative incidence of hip fracture for patients who were homozygous or heterozygous for the non-risk allele and patients homozygous for the risk allele of each single nucleotide polymorphism (analyzed by the Kaplan-Meier method).** Homozygous for the risk allele of rs2282679 (C) in the *GC* locus, a serum 25(OH)D-linked genetic variant, was significantly associated with the occurrence of hip fracture.

A multivariate Cox proportional hazards regression model adjusted for the independent non-genetic risk factors indicated that homozygous for the risk allele of rs2282679 in the *GC* locus regarding low serum 25(OH)D concentration was significantly associated with the occurrence of hip fracture (hazard ratio (95% CI) = 2.52 (1.05, 6.05), *P* = 0.039) (Table 3, Figure 2). The association was still significant when the use of active vitamin D or bisphosphonate were included in the analysis as independent variables. The proportion of patients treated with active vitamin D or bisphosphonate did not differ significantly between homozygous for the risk allele of rs2282679 (CC) and the others (data not shown). Supplemental results of the Cox regression analyses for other SNPs that were not associated with serum 25(OH)D concentration did not show a significant association with the occurrence of hip fracture (Table 3, Figure 2).

**Table 3:** Hip fracture risk for rs2282679 (GC) and the other SNPs adjusted for independent non-genetic risk factors

**Locus**	**SNP**	**HR**	**95% CI**	***P*-value**
*GC*	rs2282679	2.52	1.05, 6.05	0.039
*DHCR7/NADSYN1*	rs3829251	1.00	0.39, 2.56	1.00
	rs12785878	0.71	0.22, 2.30	0.56
	rs1790349	1.04	0.40, 2.65	0.94
*CYP2R1*	rs10741657	1.31	0.58, 3.02	0.51

All analyses were adjusted for independent non-genetic factors: Japanese version of the Health Assessment Questionnaire disability score, age, history of total knee replacement, and body mass index [16]. SNP, single nucleotide polymorphism; HR, hazard ratio; *GC*, group-specific component; *DHCR7/NADSYN1*, 7-dehydrocholesterol reductase/nicotinamide-adenine dinucleotide synthetase 1; *CYP2R1*, cytochrome P450, family 2, subfamily R, polypeptide 1.

## Discussion

In this study, an association between a polymorphism of rs2282679 in the *GC* locus and serum 25(OH)D concentration was validated in Japanese patients with RA. Minor alleles of rs2282679 had additive effects on decreasing serum 25(OH)D concentrations. In addition, rs2282679 was significantly associated with the occurrence of hip fracture in Japanese patients with RA. This is the first report that a SNP P7 in the *GC* locus was associated with the risk for hip fracture.

The *GC* gene encodes the group-specific component known as the vitamin D binding protein (DBP) that plays an important role in the vitamin D metabolic pathway [25]. Most circulating vitamin D metabolites are bound to DBP to be transported to target organs. In the previous candidate gene studies and the recent GWAS, some *GC* polymorphisms were associated with serum 25(OH)D concentration, and the strongest association was observed for rs2282679 [19,20,26]. Our results provide supportive evidence that serum 25(OH)D concentration might partly be affected by a polymorphism of rs2282679 or the other variants that are in tight linkage disequilibrium with rs2282679.

Vitamin D is an important factor in mineral metabolism, bone growth and maintenance of the skeleton [1]. In addition, 1,25(OH)_2_D, one of the vitamin D metabolites, has direct action on muscle strength and function modulated by vitamin D receptors expressed in human muscle tissue [8]. Therefore, vitamin D deficiency can lead to low bone density and muscle weakness, resulting in falls and fractures [8,27,28]. In many studies, vitamin D supplementation has been reported to reduce the risk for falls and fractures among older individuals [29,30].

To date, many genetic factors associated with low-trauma fracture including hip fracture have been reported in genome-wide meta-analysis studies [31,32]. Many variants with small effects may contribute to fracture risk, but only a few vitamin D-related genetic polymorphisms have been reported to be associated with fracture risk [33]. We explored the genetic risk of hip fracture in variants demonstrated to be associated with lower serum vitamin D concentration and found an association between rs2282679 in *GC* and the occurrence of hip fracture. Our results indicated that the risk allele carriers of the *GC* gene polymorphism tend to have low vitamin D levels that lead to greater risk of hip fracture.

Though *DHCR7/NADSYN1* and *CYP2R1* polymorphisms were associated with serum 25(OH)D concentration in the recent GWAS, we could not validate such an association in this study [19,20]. There are a number of possible explanations for the lack of an association. One is the insufficient statistical power to validate the associations. The number of samples was smaller than in the previous reports [20]. The SNP with highest statistical power to validate the association in this study was rs2282679 in the *GC* locus with a value of 0.72, and the others had comparatively lower statistical power (for example, 0.22 with rs3829251 in the *DHCR7/NADSYN1* locus). Another reason for the lack of an association is that all studied subjects were RA patients, whereas the recent GWAS were in healthy individuals [19,20]. Vitamin D is related to immunological processes, and vitamin D status has been reported to be associated with the risk of developing autoimmune diseases including RA [1,14]. In addition, serum vitamin D concentration has been shown to be lower in patients with greater disease activity [34]. Although the disease activity of the patients might affect the results of this study, DAS28 was not associated with serum 25(OH)D level in the studied population (data not shown). The difference in the genetic background between Caucasian and Japanese populations might also affect the results, which suggests genetic heterogeneity in *NADSYN1*, *DHCR7* and *CYP2R1.*

The strength of this study is that the datasets were relatively large and based on a single-institution cohort study of Japanese patients with RA. Serum 25(OH)D concentration was measured in the same season of the same year. Therefore, the differences between regions, heterogeneity and seasons had less influence on the results.

The potential limitation of this study is that the serum 25(OH)D concentration data were available from only 899 of the 1,957 patients with DNA samples, and the study on serum 25(OH)D concentration was a cross-sectional study. The smaller sample size reduced the statistical power to detect minor effects on events. Though the measurement from multiple time points would provide more valid estimates of the results, there was only one blood sample assayed for serum 25(OH)D concentration for each person. Further studies are required to confirm these associations.

## Conclusion

In conclusion, our data demonstrated that rs2282679 in *GC* was associated with both serum 25(OH)D concentration and the occurrence of hip fracture in Japanese patients with RA. These results might contribute to a better understanding of the biological impact of genetic variation within the vitamin D metabolic pathway.

## Abbreviations

25(OH)D: 25-hydroxyvitamin D; ALP: Alkaline phosphatase; BMD: bone mineral density; BMI: body mass index; CYP2R1: Cytochrome P450, family 2, subfamily R, polypeptide 1; DAS28: disease activity score in 28 joints; DBP: Vitamin D binding protein; DHCR7/NADSYN1: 7-dehydrocholesterol reductase/nicotinamide-adenine dinucleotide synthetase 1; DMARD: Disease modifying antirheumatic drug; GC: Group-specific component; GWAS: Genome-wide association studies; IORRA: Institute of Rheumatology, Rheumatoid Arthritis; IQR: inter-quartile range; J-HAQ: Japanese version of the Health Assessment Questionnaire; MAF: minor allele frequency; NSAID: non-steroidal anti-inflammatory drug; RA: rheumatoid arthritis; RF: rheumatoid factor; SE: standard error; SNP: single nucleotide polymorphism; TKR: total knee replacement.

## Competing interests

The authors declared that they have no competing interests.

## Authors’ contributions

KI designed the study. KI, TF, YT, AT, HY and SM collected DNA samples and the data on fracture. SY and KI performed genotyping. SY and KI contributed to the statistical analyses. SY and KI wrote most of the manuscript. All authors contributed to writing and correcting the manuscript and have approved the final version.
